# Generation of lachrymatory factor synthase-suppressed onion (*Allium cepa* L.) by *Agrobacterium*-mediated gene transfer for CRISPR/Cas9 genome editing

**DOI:** 10.3389/fpls.2026.1949452

**Published:** 2026-09-30

**Authors:** Shoya Tamaru, Shinsuke Imai, Satoshi Watanabe, Tatsuya Ikegai, Sachie Kondo, Tomoko Igawa, Takahiro Kamoi

**Affiliations:** 1 Basic Research Division, Research & Development Headquarters, House Foods Group Inc., Chiba, Japan; 2 Graduate School of Horticulture, Chiba University, Chiba, Japan; 3 Plant Molecular Science Center, Chiba University, Chiba, Japan; 4 Research Center for Space Agriculture and Horticulture, Chiba University, Chiba, Japan

**Keywords:** *Agrobacterium*, CRISPR/Cas9, genome editing, lachrymatory factor synthase, onion transformation

## Abstract

**Introduction:**

Lachrymatory factor, an irritating volatile compound that induces tearing, is produced when onion bulbs are cut or chopped. Lachrymatory factor synthase (LFS)-specific suppression in onion may reduce the lachrymatory effect and enhance flavor and health-functional properties. Genome editing enables precise modification of target genes in plant genomes. However, no reports targeting genes controlling onion-specific traits have been published. Therefore, we aimed to generate onion plants with reduced LFS activity via clustered regularly interspaced short palindromic repeats (CRISPR)/CRISPR associated protein 9 (CRISPR/Cas9) genome editing.

**Methods:**

Calli induced from primary roots were transformed with *Agrobacterium tumefaciens* carrying expression cassettes for CRISPR/Cas9, guide RNA, green fluorescent protein (GFP), and hygromycin resistance; callus lines with a high frequency of stable GFP expression were selected as “elite callus lines” suitable for transformation. Cleaved amplified polymorphic sequence (CAPS), heteroduplex mobility assay (HMA), and Sanger sequencing confirmed mutations introduced into the *LFS* gene, and plants were regenerated from the confirmed *LFS*-edited callus lines.

**Results and discussion:**

The LFS enzyme activity was lower in the leaves and bulbs of the *LFS*-edited plants than in those of control plants, while the *LFS*-edited plants exhibited severe growth abnormalities and failed to set seed, possibly due to long-term culturing to maintain the elite callus line. The present study demonstrates the feasibility of onion genome editing to modify a specific onion trait, namely, LFS activity. Our findings provide a basis for further development toward the ultimate goal of producing tear-free onions with enhanced health-functional properties.

## 1 Introduction

Onion (*Allium cepa* L.) is an important vegetable crop, ranking second in global production after tomato (FAOSTAT, 2024). One of its notable characteristics is the lachrymatory effect that occurs when the bulbs are cut. The lachrymatory factor (LF), a volatile compound produced enzymatically from sulfenic acids, is synthesized by lachrymatory factor synthase (LFS) (**Figure 1**) (Imai et al., 2002). In nature, this compound and its rapid enzymatic generation system are considered to function as a chemical defense mechanism against herbivores and pathogens. Low-lachrymatory onions with reduced LFS activity have been generated by RNA interference (RNAi) (Eady et al., 2008). A previous study showed that LFS suppression not only reduced the lachrymatory effect but also increased the amount of thiosulfinates, potentially contributing to enhanced health-functional properties and flavor. In our previous study, we established a model experimental system for evaluating onion bulb components using LFS-suppressed onions in comparison with normal onions (Eady et al., 2008). This system can estimate LFS activity from experimentally generated and quantified LF using the following procedures: sufficient alliinase is added to the substrate *E*-(+)-S-(1-propenyl)-L-cysteine sulfoxide (PRENCSO), and the generated intermediate 1-propenesulfenic acid is reacted with an onion reaction mixture containing LFS. Furthermore, the model system has enabled the identification of the structures of thiosulfinates and sulfides that were elevated upon LFS suppression and revealed that some of these compounds possess inhibitory activity against enzymes such as cyclooxygenase-1 (COX-1), providing health-promoting effects (Aoyagi et al., 2011, 2021). Another tear-free onion has been produced through mutagenesis induced by a heavy-ion beam (Kato et al., 2016). In the mutant lines, LF as well as pyruvic acid were reduced due to repression of expression of the gene encoding alliinase, suggesting that thiosulfinate, which is involved in health-promoting effects, was also reduced (**Figure 1**).

**Figure 1:**
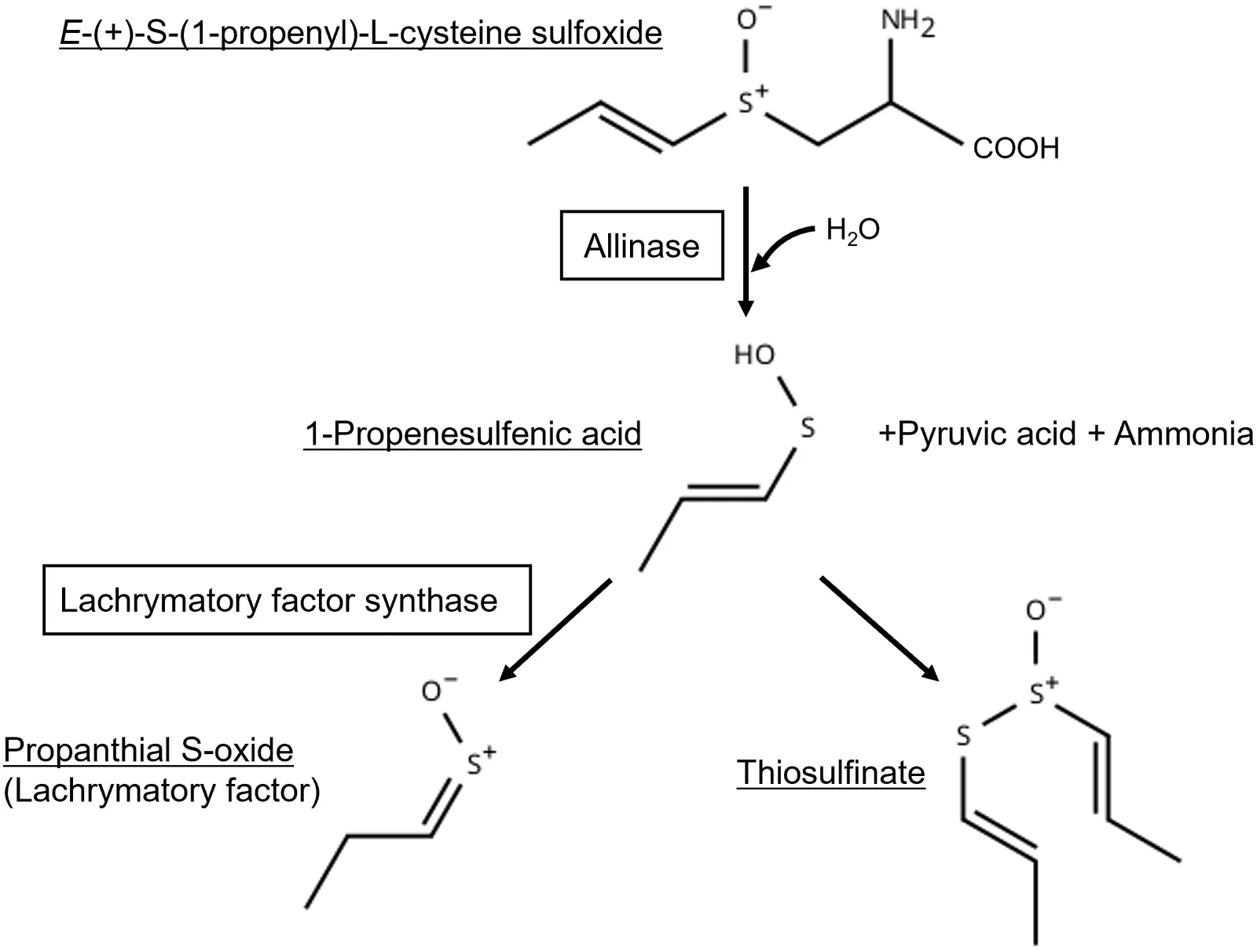
Biosynthetic pathway of lachrymatory compounds and thiosulfinates in onion. Alliinase catalyzes the conversion of the substrate *E*-(+)-S-(1-propenyl)-L-cysteine sulfoxide (PRENCSO) to generate the intermediate 1-propenesulfenic acid, along with pyruvic acid and ammonia. Subsequently, lachrymatory factor (LF) synthase (LFS) catalyzes the conversion of this intermediate to produce LF. Furthermore, thiosulfinates, which are involved in health-functional properties, are generated spontaneously.

Our research group has conducted detailed, long-term analyses of onion-specific sulfur-containing compound metabolism and health-functional properties, with a particular focus on LFS. Based on these studies, LFS-specific suppression in onion is expected to reduce the lachrymatory effect and enhance flavor and health-functional properties, thereby improving suitability for raw consumption. However, onions with low LFS activity have not yet been generated by conventional cross-breeding nor identified as naturally occurring variants.

Considering the biosafety and regulatory framework under the Cartagena Protocol, genome editing is a promising approach for precision breeding. In recent years, technologies such as clustered regularly interspaced short palindromic repeats (CRISPR)/CRISPR-associated protein 9 (CRISPR/Cas9) have been developed, enabling precise modification of target genes in plant genomes. However, onion has been recognized as one of the most recalcitrant crops for genetic transformation and genome editing due to its low transformation efficiency and strong genotype dependence. Consequently, only a few reports are available. While successful genome editing of the phytoene desaturase (*PDS*) gene in onion has been reported (Mainkar et al., 2023), to our knowledge, no reports targeting genes controlling onion-specific traits have been published.

Therefore, we aimed to generate onion plants with reduced LFS activity via CRISPR/Cas9 genome editing. This study provides aninitial report of the generation of an onion line with a reduced lachrymatory effect and increased flavor and health-functional components through genome editing of the *LFS* gene.

## 2 Materials and methods

### 2.1 Plant material

An onion cultivar ‘Senshu-Chu-Koudakaki’, an open-pollinated variety, was used in this study. *In vitro* seedlings were cultured under 12 h light/dark at 25 °C to reach a height of at least 15 cm. Bulb formation was then promoted by further culturing under 14/10 h light/dark at 25 °C. Alternatively, large seedlings grown *in vitro* were transferred to soil, and bulb enlargement was attempted following acclimatization. For acclimatization, plants transferred to soil were covered with plastic wrap to maintain humidity and were gradually adapted to external environmental conditions over a period of 1 week. After transfer to soil, plants were cultivated under 14/10 h light/dark at 25 °C.

### 2.2 Construction of CRISPR/Cas9 vectors

Seven guide RNAs (gRNAs) (#1, #2, #3, #4, #5, #6, and #7) were designed to target specific sequences proximal to the translation initiation site or the enzymatically active sites within the *LFS* open reading frame (ORF) (GenBank accession no. AB089203.1). To evaluate and select the optimal spacer sequences, in silico gRNA design tools such as E-CRISP (Heigwer et al., 2014) and WU-CRISPR (Wong et al., 2015) were utilized. The positions of the target sites and PCR primers are shown in **Figure 2**, and their nucleotide sequences are listed in **Supplementary Tables 1** and **2**.

**Figure 2:**
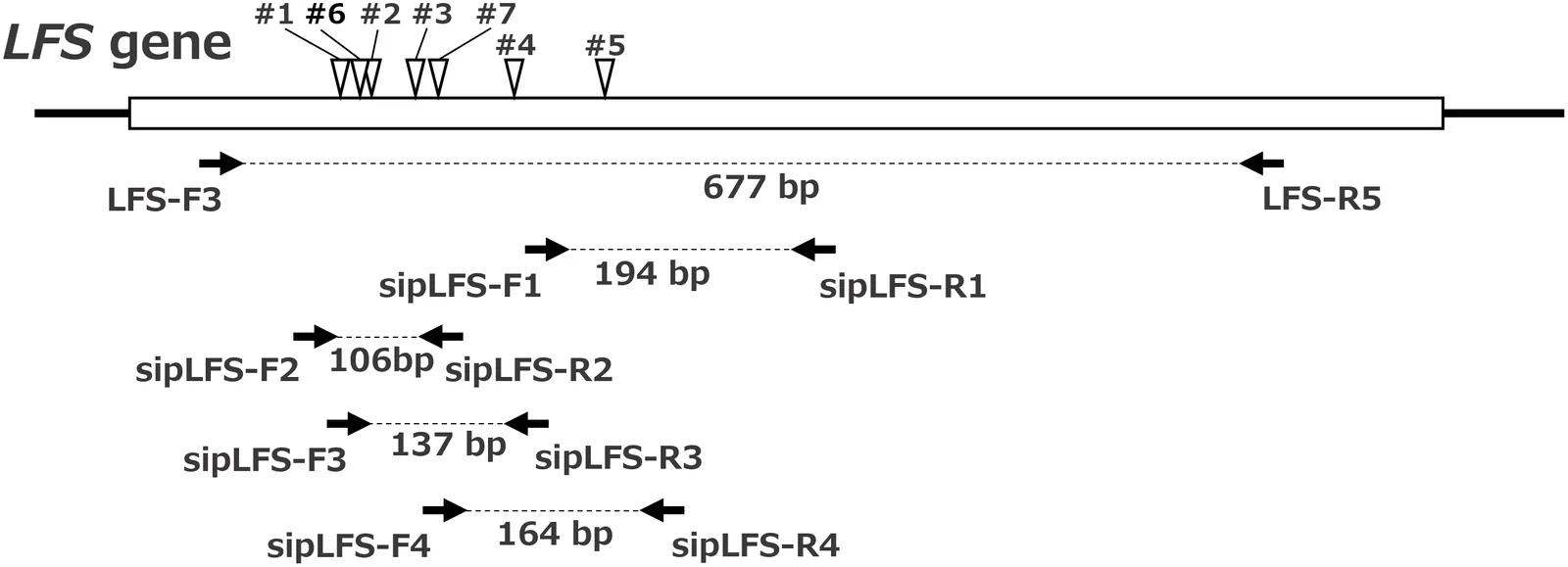
Positions of target sequences (triangles) and polymerase chain reaction (PCR) primers (arrows) within the *LFS* gene (accession no. AB089203.1). The amplicon size (bp) for each primer pair is indicated under the dashed line. Black vertical lines and a white box represent the untranslated regions and the intronless open reading frame (ORF) of *LFS*, respectively. Detailed nucleotide sequences of the targets and primers are listed in **Supplementary Tables 1** and **2**, respectively.

Two CRISPR/Cas9 vectors were constructed: pBI_Cas9-gRNA_HygR_GFP (carrying a single gRNA) (**Figure 3A**) and pBI_Cas9-gRNA_+3gRNA_HygR_GFP (carrying four gRNAs) (**Figure 3B**). The multiplex vector was employed because simultaneous introduction of multiple gRNAs with the dMac3 enhancer has been reported to improve editing efficiency (Kusano et al., 2018). The binary vector pBI-GW-NOS (Inplanta Innovations Inc., Yokohama, Japan) was used as the backbone. For both binary vectors, Cas9 and gRNA expression was driven by the mannopine synthase (MAS) promoter, which enables bidirectional transcription (Leung et al., 1991). To enhance Cas9 translation, the duplicated MAP-kinase activating protein 3 (dMac3) sequence (Aoki et al., 2014) was used. As selectable markers, the hygromycin resistance gene (*HygR*) and enhanced green fluorescent protein (EGFP) were driven by the nopaline synthase (NOS) and cauliflower mosaic virus (CaMV) 35S promoters, respectively. The NOS terminator was used for all expression cassettes based on a previous successful onion transformation using multiple NOS terminators (Kamata et al., 2011). The overall T-DNA structures of the two CRISPR/Cas9 vectors are shown in **Figure 3**.

**Figure 3:**
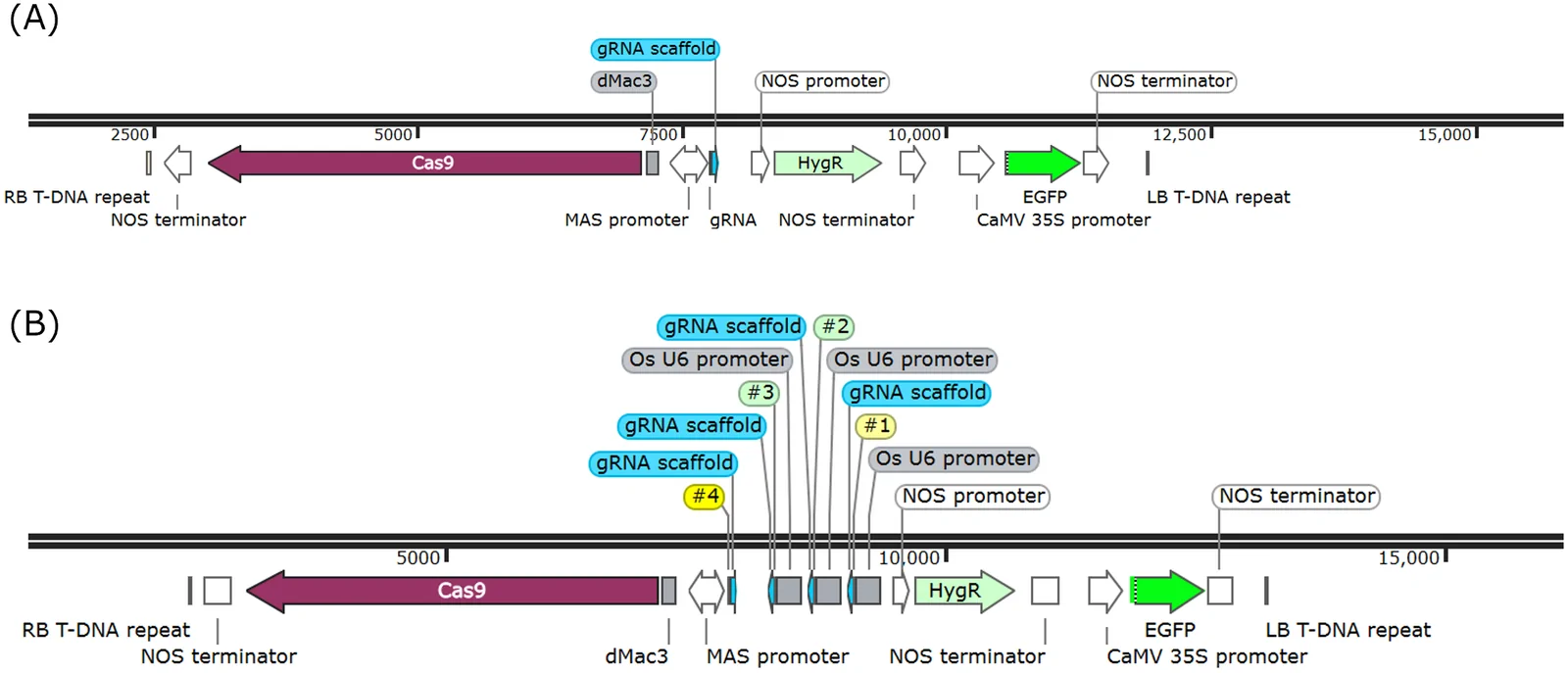
T-DNA structures of the CRISPR/Cas9 vectors. **(A)** pBI_Cas9-gRNA_HygR_GFP, carrying a single gRNA (#4, #5, #6, #7 each). **(B)** pBI_Cas9-gRNA_+3gRNA_HygR_GFP, carrying four gRNA scaffolds (#1, #2, #3, and #4). LB, left border; RB, right border; NOS, nopaline synthase; dMac3, duplicated Mac3 translational enhancer; MAS, mannopine synthase; OsU6, Oryza sativa U6; HygR, hygromycin resistance gene; CaMV, cauliflower mosaic virus; EGFP, enhanced green fluorescent protein.

The constructed plasmids were introduced into *Agrobacterium tumefaciens* strain LBA4404 as described previously (Ditta et al., 1980). *A. tumefaciens* culture and callus infection were performed as described previously (Kamata et al., 2011), with a minor modification; carbenicillin (250 mg•L^-1^) was used instead of cefotaxime (500 mg•L^-1^) in the selection and regeneration media. The compositions of the media used in the experiments are listed in **Figure 4B**.

**Figure 4:**
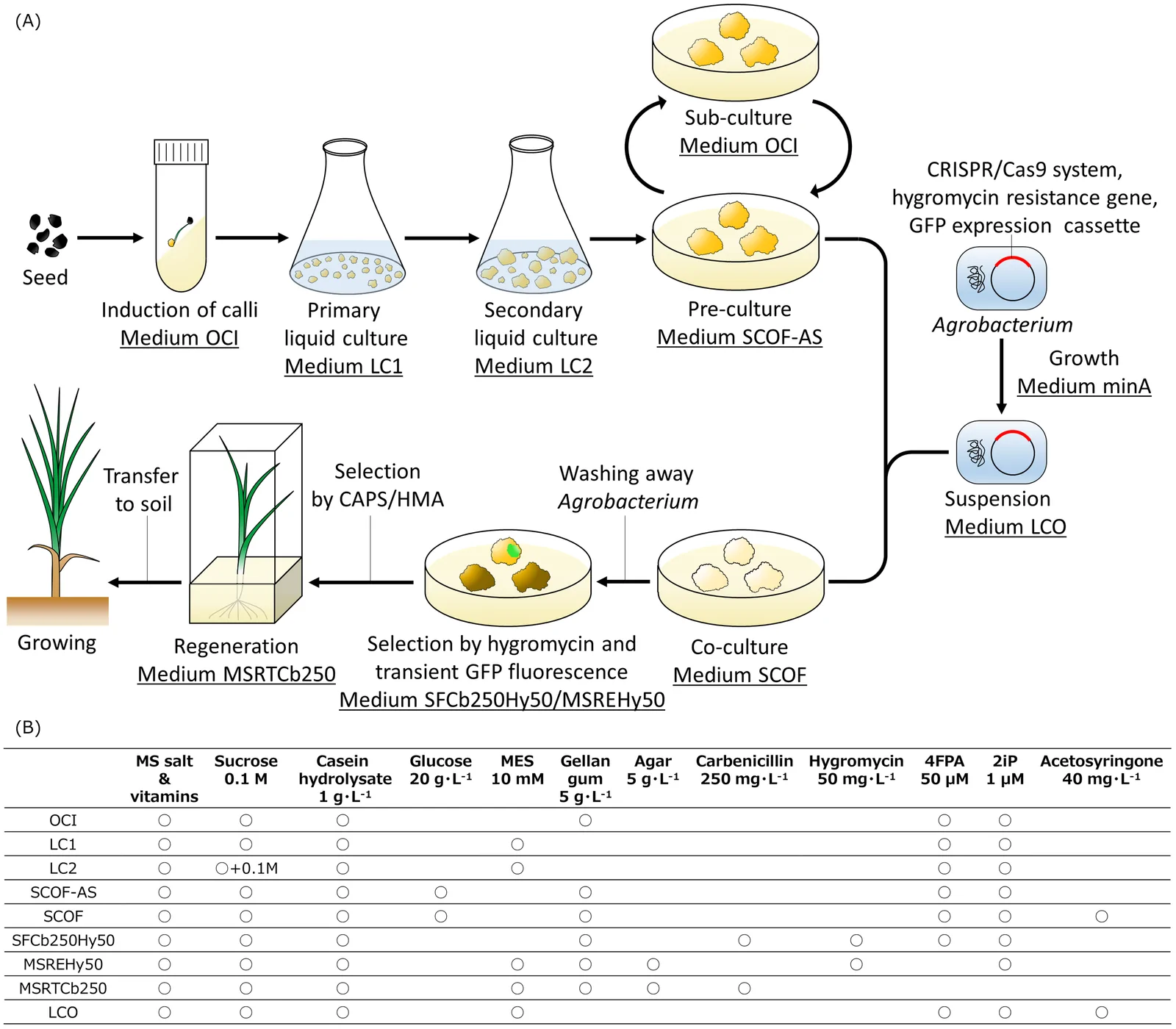
Transformation procedures and culture media for generating LFS-edited onion plants. **(A)** Overview of the transformation and selection steps. GFP, green fluorescent protein; CAPS, cleaved amplified polymorphic sequence; HMA, heteroduplex mobility assay. **(B)** Composition of the culture media. MS, Murashige and Skoog medium; MES, 2-morpholinoethanesulphonic acid; 4FPA, 4-fluorophenoxyacetic acid; 2iP, N^6^(2-isopentenyl)adenine.

### 2.3 Elite callus preparation

Callus induction and preparation for *Agrobacterium* infection were performed as described previously (Kamata et al., 2011). An overview of the procedure and the culture media used at each stage of culture is shown in **Figure 4A**. The cultured *A. tumefaciens* cell suspension LBA4404 was prepared in minA solution (2.1 g •L^-1^ K_2_HPO_4_, 0.9 g •L^-1^ KH_2_PO_4_, 0.2 g •L^-1^ (NH_4_)_2_SO_4_, 0.1 g •L^-1^ trisodium citrate dihydrate, 0.2 g •L^-1^ MgSO_4_·7H_2_O, 2 g•L^-1^ glucose, and 400 mg •L^-1^ kanamycin). The compositions of all other media used in this study are listed in **Figure 4B**. For the selection of elite callus lines, *Agrobacterium* that harbored a binary vector constructed using pBI-GW-NOS as the backbone vector, which carries a GFP expression cassette, was used for infection. The two vectors shown in **Figure 3** were also used in the evaluations. After 1 month of culture, stable GFP expression was observed as described in Section 2.4. The callus line exhibiting a higher and reproducible rate of GFP expression was selected as the elite line.

### 2.4 Microscopy

GFP signal was observed using a Nikon SMZ18 fluorescence stereomicroscope with a filter cube (P2-EFL GFP-B or P2-EFL GFP-L) (Nikon Solutions, Co., Ltd., Tokyo, Japan). Calli with detectable GFP signal 1 month after transformation were used as transgenic plants for subsequent genetic analyses.

### 2.5 *LFS* mutation analysis of transgenic calli and plant lines

Genomic DNA (gDNA) was extracted from calli, leaves, and bulbs of regenerated plants using a DNeasy Plant Mini Kit (QIAGEN, Hilden, Germany). When a suitable restriction enzyme recognition site was present or absent in the target sequence, cleaved amplified polymorphic sequence (CAPS) or heteroduplex mobility assay (HMA) was performed using primers listed in **Supplementary Table 2**. For both analyses, the nested polymerase chain reaction (PCR) products amplified from the LFS-F3/LFS-R5 amplicon were used. For the CAPS assay, the PCR products were digested with specific restriction enzymes (*MseI* for #5, *SacI* for #6, and *XcmI* for #7). For the HMA, the heteroduplexes in the PCR products were examined using the MultiNA microchip electrophoresis system (Shimadzu Corporation, Kyoto, Japan).

Samples that exhibited extra bands or mobility shifts, compared with the wild-type (WT), were selected as mutation candidates. To qualitatively confirm the mutations in these candidates, mutation patterns were detected by Sanger sequencing. For the quantitative determination of editing rates, next-generation sequencing (NGS) was performed on a partial LFS sequence amplified using the primer set LFS-F3/LFS-R5 (**Figure 2**; **Supplementary Table 2**) using KOD FX DNA polymerase (TOYOBO Co., Ltd., Osaka, Japan) at an annealing temperature of 55 °C. Mutation frequencies were calculated as the proportion of reads that carried mutations among the total number of reads (approximately 50,000–80,000 reads per plant).

### 2.6 LFS activity measurement

LFS activity was measured as described by Eady et al. (2008). To homogenize the samples, the harvested *in vitro*-grown leaves or bulbs were freeze-dried and ground into a powder. An equal amount of each freeze-dried powder was suspended in water and centrifuged (20,400 ×g, 5 min, 4 °C). The resulting supernatant was used as the LFS enzyme extract. Protein concentration was determined using a BCA Protein Assay Kit (Thermo Fisher Scientific, Waltham, MA, USA), according to the manufacturer’s instructions. Protein concentrations among the samples were adjusted to the same level, and relative LFS activity for each extract was measured.

## 3 Results

### 3.1 Selection of elite callus lines

We found that stable GFP expression rates differed among callus lines derived from individual seeds, even within the same open-pollinated variety (‘Senshu-Chu-Koudakaki’), indicating strong genotypic dependence (**Table 1**). While substantial variation in stable GFP expression levels was noted among experimental lots, callus line 130 exhibited the highest transformation efficiency (86.3%) among the generated lines (**Table 1**). Although reducing totipotency during long-term maintenance of callus cultures is a well-known phenomenon, we confirmed that the regenerative capacity of these lines was maintained for more than 1 year through subculturing on OCI medium (**Figures 4B**, **5**). Based on these results, we selected an elite callus line that exhibited a high rate of GFP expression for subsequent Agrobacterium infection.

**Table 1:** Stable green fluorescent protein (GFP) expression rate in different callus lines.

Callus line	Experiment no.							
	1		2		3		4	
130	59.4%	(329/554)	20.5%	(61/297)	86.3%	(113/131)	66.0%	(157/238)
132	12.9%	(60/465)	9.8%	(49/499)	–	–	–	–
134	20.0%	(55/275)	28.7%	(80/279)	–	–	–	–
136	17.9%	(122/683)	41.3%	(71/172)	–	–	–	–
145	7.1%	(39/553)	18.4%	(73/397)	4.8%	(6/126)	4.2%	(12/285)
147	8.2%	(29/353)	10.6%	(91/857)	2.0%	(3/150)	3.4%	(11/326)

**Figure 5:**
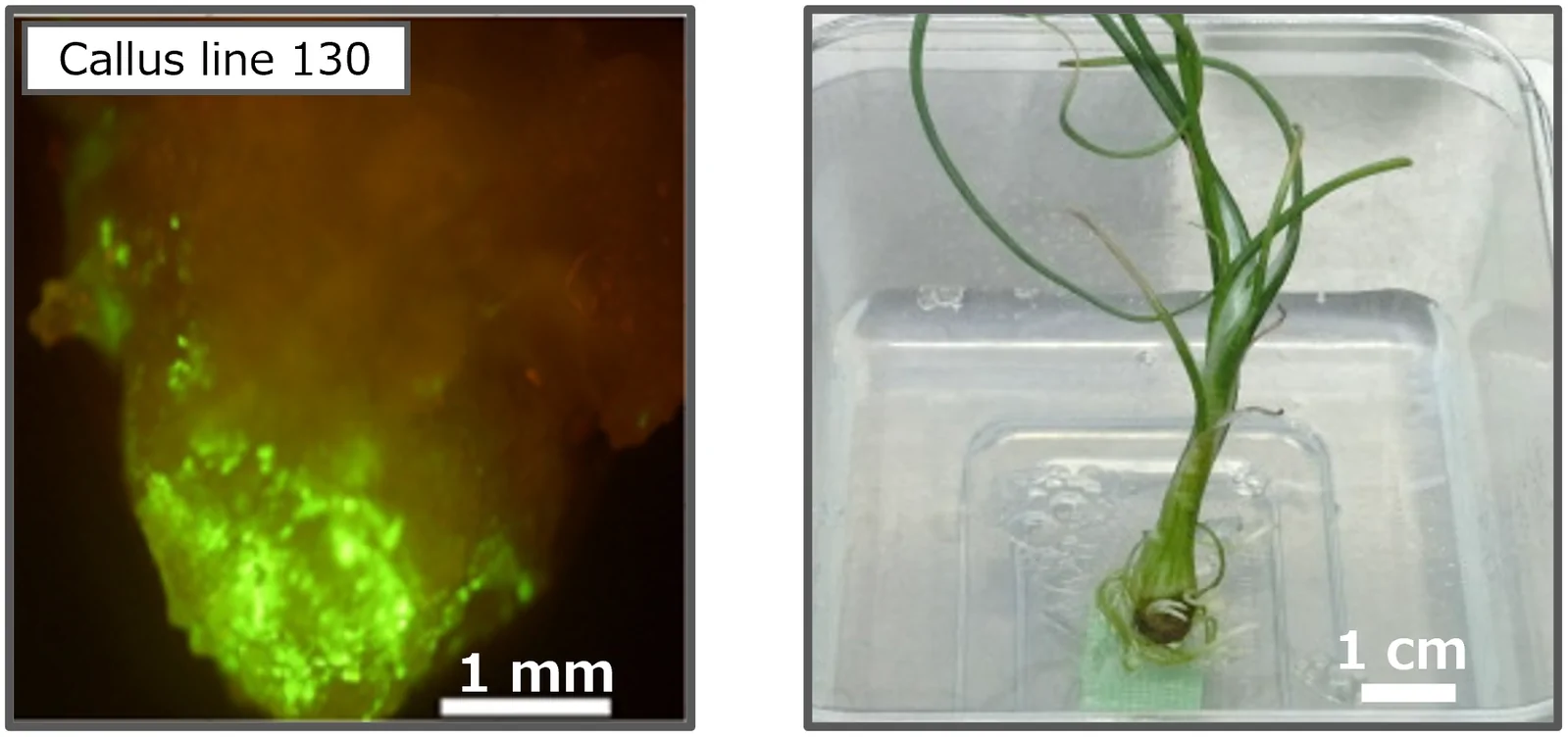
Stable green fluorescent protein (GFP) expression (left) and a regenerated plant (right) derived from callus line 130. Stable GFP expression was observed 1 month after *Agrobacterium* infection.

### 3.2 *LFS* mutation analysis of the transgenic calli and regenerated plants

The results of genome editing by five different gRNAs are summarized in **Table 2**. Among 483 transgenic callus lines that stably expressed GFP, *LFS* mutations were detected by CAPS and HMA analyses in 101 lines. Consequently, among the single gRNA expression cassettes, mutations were detected in callus lines targeted by gRNAs #4, #5, or #7 but not by #6. In the case of the quadruple gRNA expression cassette targeting #1, #2, #3, and #4 simultaneously, editing was successfully induced at three of the four target sites (#1, #2, and #4), whereas no mutations were detected at #3 site.

**Table 2:** Clustered regularly interspaced short palindromic repeats (CRISPR)/CRISPR associated protein 9 (CRISPR/Cas9)-induced mutations in callus lines and regenerated plants by guide RNA (gRNA).

Target	No. of callus		No. of regenerated plants	
	Analyzed	Edited	Analyzed	Edited
#1, 2, 3, 4	55	22	15	4
#4	120	28	7	7
#5	17	4	0	0
#6	33	0	0	0
#7	258	47	8	8

In total, 71 regenerated plants were obtained from the mutation-positive callus lines, while no plants were obtained from the calli with mutations in #5 target sequence. Of the regenerated plants, 30 plant lines that exhibited normal growth were subjected to NGS analysis to characterize the mutation patterns and frequencies (**Table 3**). Consequently, *LFS* mutations were confirmed in 19 regenerated plant lines. Among them, five plant lines (designated with the prefix “P” for plant; P2, P20, P40, P130, and P464) with mutation frequencies >50% were identified, and 3–7 mutation patterns were detected in each line (**Table 3**; **Figures 6**, **7A**).

**Table 3:** Number of *LFS*-mutated plants and their mutation frequencies.

	No. of LFS-edited plants									
	Mutation frequencies by NGS (%)									
Target	0–10	10–20	20–30	30–40	40–50	50–60	60–70	70–80	80–90	90–100
#1, 2, 3, 4	11	–	–	1	–	–	1	–	1	1
#4	–	–	3	2	1	1	–	–	–	–
#7	–	4	2	1	–	1	–	–	–	–

Target sequences included in the guide RNA (gRNA) are shown in the left columns. The number of plants showing each mutation frequency range determined by next-generation sequencing (NGS) is indicated in the table. The number of plant lines with a mutation frequency of more than 50% is highlighted in red. LFS, lachrymatory factor synthase.

**Figure 6:**
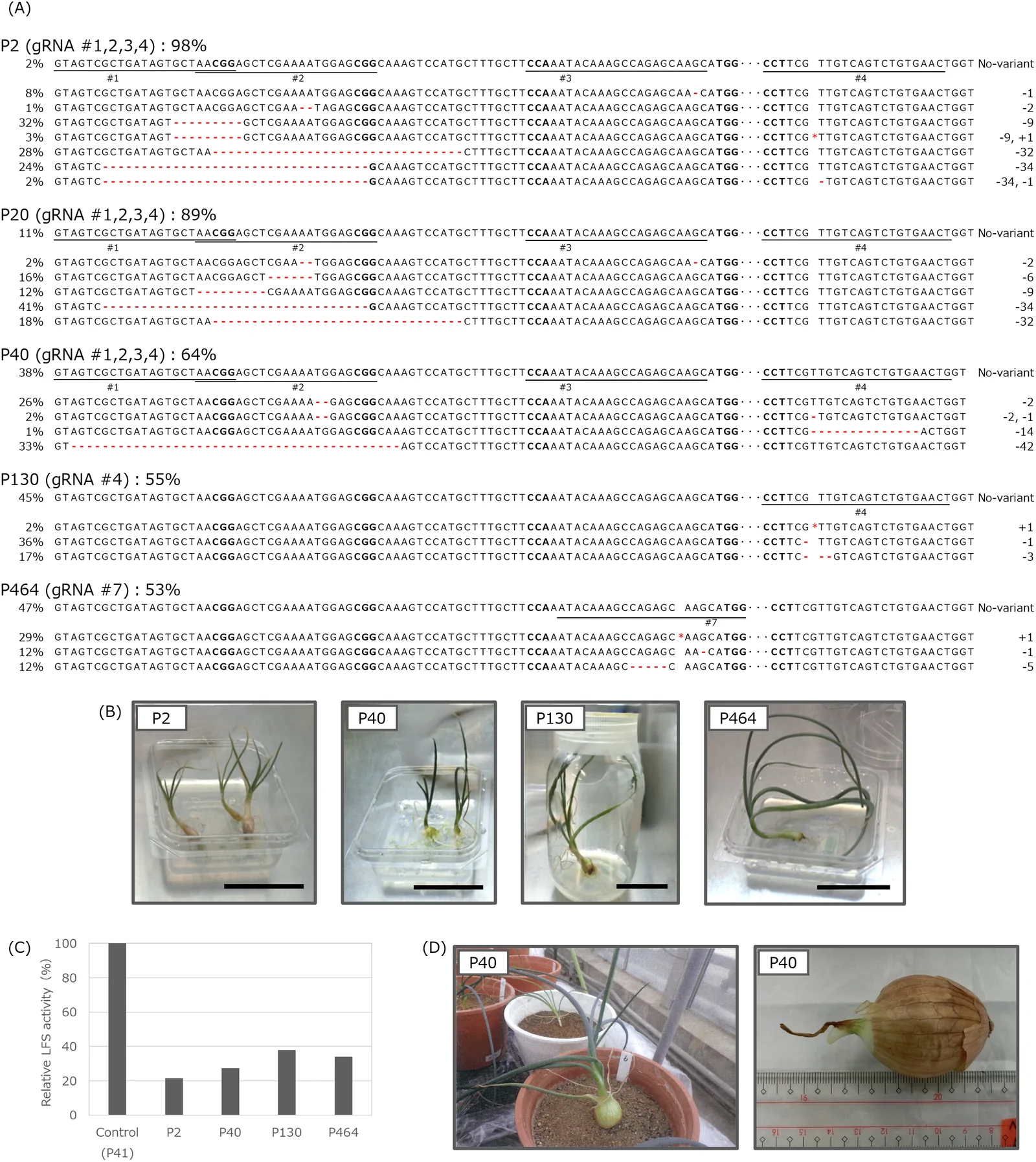
*LFS* mutant *(lfs*) plant lines and their lachrymatory factor synthase (LFS) enzyme activities. **(A)** Mutation patterns and frequencies in each *lfs* plant line (T_0_). Target sequences are underlined. Bold nucleotides within each target indicate the protospacer adjacent motif (PAM) sequence. Red hyphen (-) and asterisk (*) denote nucleotide deletions and insertions, respectively. The numbers on the right indicate the number of inserted (+) and/or deleted (-) nucleotides detected in each sequence. **(B)** *In vitro*-grown regenerated plant lines P2, P40, P130, and P464 (from left to right). Bar = 5 cm. **(C)** Relative LFS enzyme activity in the leaves of the *lfs* plant lines. The average activity (%) from two technical replicates (independent extractions from the same T_0_ plant) for each sample is indicated. Error bars are not shown due to the small number of replicates (n = 2). **(D)** Growth of plant line P40 during soil cultivation (left) and the harvested bulb (right).

**Figure 7:**
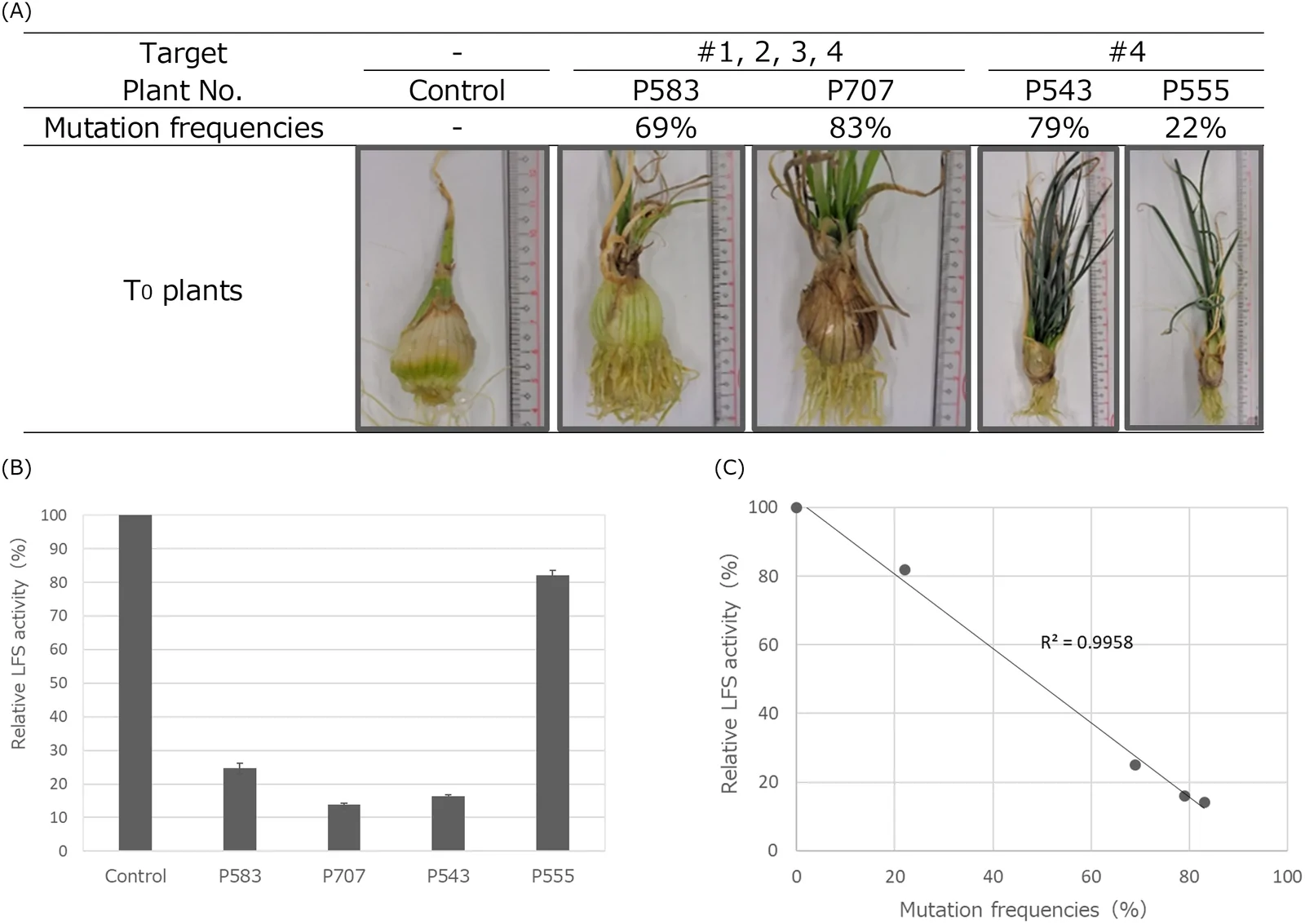
*In vitro* bulb formation for evaluation of lachrymatory factor synthase (LFS) activity. **(A)** *In vitro*-formed bulbs of the *lfs* plants and their morphologies. Targets for obtaining the genome-edited plants are indicated at the top of each photo. The mutation frequencies determined by next-generation sequencing (NGS) are indicated below the T_0_ plant numbers. **(B)** Relative LFS activity in the bulbs of T_0_ *lfs* plant lines. The average relative activity (%) from three technical replicates (independent extractions from the same T_0_ plant) for each sample is indicated. The vertical bars on each graph represent the standard deviation of the technical replicates. **(C)** Relationship between mutation frequency and relative LFS activity in bulbs from T_0_ *lfs* plants. The point at 0% mutation frequency/100% LFS activity is derived from the non-transgenic control.

### 3.3 LFS enzyme activity in the leaves of *lfs* plants

LFS activities in the leaves of the four mutant plant lines (P2, P40, P130, and P464) were measured (**Figure 6C**). P20 was excluded from this assay because it died prior to the measurement. Initially, we attempted to use a non-transformed regenerated plant as a WT control; however, it died due to poor growth during cultivation. Plant P41 was derived from a transgenic callus line introduced with gRNAs for #1, #2, #3, and #4. Although it was a transgenic plant that carried the T-DNA, NGS confirmed that it remained unedited at the *LFS* locus; therefore, it was used as the experimental control instead. Consequently, relative LFS activities in all four *lfs* plant lines were reduced to <40% of that in the control plant P41. The greatest reduction in activity was detected in P2 (21.5% relative to that of the control), which also had the highest mutation frequency (**Table 3**; **Figure 6A**). Furthermore, a correlation between the mutation frequencies and the reduction in LFS activity in the leaves was observed (*R*^2^ = 0.9164).These four *in vitro*-grown *lfs* plants were transplanted to soil to obtain sufficiently large bulbs and progeny seeds. However, all the plants exhibited considerably more retarded growth than the control seedlings, and P2, P130, and P464 eventually died. Plant P40 toppled over during cultivation; therefore, the bulb had to be harvested when it was approximately 20 mm in diameter, which was too small (**Figure 6D**). Consequently, the bulbs subjected to vernalization died during cold storage due to rot, and no progeny seeds were obtained.

### 3.4 LFS enzyme activity in freeze-dried bulbs derived from *lfs* plants

As soil cultivation of P2, P40, P130, and P464 plants was unsuccessful, we attempted *in vitro* bulb formation instead, initially aiming to obtain larger bulbs suitable for subsequent seed production. We obtained four new T_0_ *lfs* plant lines—P543, P555, P583, and P707 (**Figure 7A**), using the procedures described above. These *in vitro* plants were cultured in large vessels containing MSREHy50 medium (**Figure 4B**) under long-day conditions (18/6 h light at 24 °C/20 °C). All the newly obtained *lfs* plants successfully formed bulbs, and P707 in particular produced a bulb approximately 30 mm in diameter and weighing approximately 10 g (**Figure 7A**), although the bulb sizes were not comparable to those typically produced by WT onions under conventional cultivation. As the *in vitro*-formed bulbs did not reach a sufficient size for seed production, we ultimately decided to use them for LFS activity assays. In this experiment, control plants regenerated from non-transgenic calli were also cultured under identical conditions and likewise exhibited small bulbs. In contrast, seedlings obtained directly from seeds, without a callus phase, grew and developed bulbs exceeding 100 mm in diameter under the same *in vitro* conditions.

Next, LFS activity was measured in the obtained bulbs. All samples showed lower LFS activities than those of the non-transgenic control (**Figure 7B**). A strong correlation was observed between the LFS reduction rate and mutation frequency in the bulbs (*R*^2^ = 0.9958) (**Figure 7C**).

## 4 Discussion

We generated onion plants with reduced LFS activity via CRISPR/Cas9 genome editing. The LFS enzyme activity was lower in the leaves and bulbs of the *LFS*-edited plants than in those of control plants. Overall, we demonstrate the feasibility of onion genome editing to modify a specific onion trait, the LFS activity.

### 4.1 Callus line suitable for *Agrobacterium* transformation

In *Agrobacterium*-mediated transformation experiments using onion callus, high efficiencies of both plant regeneration and transformation are important. Tanikawa et al. (1998) reported that plant regeneration efficiency from callus differs among onion cultivars; Kamata et al. (2011) reported that calli with weak autofluorescence show higher transformation efficiency than those with strong autofluorescence, even when derived from the same cultivar. Based on these findings, we used ‘Senshu-Chu-Koudakaki’ as an appropriate cultivar for the present study and selected calli that showed weak autofluorescence for transformation. We further confirmed strong genotype dependency for transformation efficiency by comparing calli derived from individual seeds. No morphological differences between calli with low and high transformation efficiencies could be distinguished at the stereomicroscopy level. Notably, elite callus lines with high GFP expression rates maintained this trait even after a long culture period with repeated subculturing. Therefore, preliminary evaluation with a visible marker is a critical step for *Agrobacterium*-mediated onion transformation. Future analyses, such as RNA-seq, proteomic analyses, and phytohormone measurements, would provide molecular and physiological insights into differences between recalcitrant and amenable cell lines, contributing to the efficient selection of elite lines.

### 4.2 Selection of an optimal target sequence for Cas9-inducible indel mutation

In this study, *lfs* mutant plants were successfully obtained by genome editing targeting *LFS*. We selected seven target sequences and designed corresponding gRNAs based on their positions relative to regions that affect translation or enzyme activity. Consequently, mutations were detected with gRNAs #1, #2, #4, #5, and #7, whereas no mutations were detected with gRNAs #3 or #6. The difference in mutation frequencies (**Figure 6A**) may depend on the sequence-dependent efficiency of Cas9-induced mutagenesis. In recent years, artificial intelligence-based gRNA design programs such as DeepSpCas9 (https://deepcrispr.info/) (Kim et al., 2019) have been developed. The DeepSpCas9 score of each target sequence used in this study showed that target sequences with scores >40 caused mutations, whereas two of the three targets with scores <40 produced no detectable mutations, except for target #5 (**Supplementary Table 3**). In particular, #4, with the highest score, generated an *lfs* mutant with a mutation frequency >50%, and the combination of gRNAs #1 #2, and #4 appeared to enhance the mutation frequency (**Table 3**). The highly complex mutation profile observed in this study is consistent with the fact that the *LFS* gene exists as a multicopy gene family in the onion genome (Masamura et al., 2012). Overall, as the results of this study showed a rough correlation between the DeepSpCas9 score and mutation efficiency, the use of such prediction tools may be effective for selecting target sequences.

### 4.3 Achievement of reduced LFS activity in *lfs* plants

Based on the previous identification of the LFS enzyme responsible for the production of lachrymatory factor, disruption of LFS function is an ideal strategy for generating tear-free onions with higher health-functional properties (Imai et al., 2002; Eady et al., 2008). Additionally, precision breeding is highly desirable. In this study, the *lfs* plant lines with mutation frequencies >50% exhibited reduced LFS activities, indicating that the proposed strategy was successful as expected.

However, unexpectedly, the *lfs* plants did not grow normally after acclimatization; therefore, bulbs of normal size and progeny seeds could not be obtained. As biological replicates could not be prepared from these seedless T_0_ plants that harbored independent mutations, the LFS activity data presented here should be considered descriptive and preliminary. The correlation between mutation frequency and LFS activity was more linear in bulbs (*R*^2^ = 0.9958) than in leaves (*R*^2^ = 0.9164). This slight discordance might reflect sampling variance in chimeric T_0_ plants or potentially tissue-specific expression patterns of the *LFS* gene.

The use of mature seeds for callus induction has an advantage in providing year-round starting material and facilitating easier manipulation, compared with the use of calli induced from immature embryos in a previous study (Eady et al., 2000). In addition, all elite callus lines subjected to long-term subculture for 3 years or longer were able to regenerate plants. However, very poor growth in soil after acclimatization resulted in severely stunted bulb development, with bulbs not exceeding approximately 4 cm in diameter. Non-transgenic plants derived from non-elite callus lines maintained for 3 years or longer also produced bulbs not exceeding approximately 4 cm in diameter (**Figure 7A**, Control). Moreover, defects in bulb formation and morphological abnormalities of the roots and leaves during soil cultivation were observed in all plants derived from different elite callus lines, which were induced from different seed genotypes. Even in the *in vitro*-grown plants that successfully formed larger bulbs (**Figure 7A**), abnormalities such as bushy leaves and poor root elongation were observed. Notably, P555 exhibited abnormal morphology despite having a low mutation frequency (22%) and retaining high LFS activity (~82%), essentially resembling the WT phenotype. This observation strongly supports the explanation that the growth defects are primarily caused by the long-term callus culture process. Furthermore, previous studies have shown that LFS suppression by RNAi (Eady et al., 2008) does not cause severe growth defects. Given these results, the poor growth observed here was unlikely to be solely attributed to off-target effects of the gRNAs, the particular seed genotype used here, or to the effects of LFS reduction. However, because a genome-edited plant generated without a callus phase is currently unavailable for comparison, the potential contributions of LFS reduction or off-target effects to the poor growth cannot be completely excluded. Somaclonal variation accompanied by epigenetic modifications may have occurred (Kaeppler et al., 2000; Debnath and Ghosh, 2022), although the causes of poor growth under soil cultivation remain unclear. In onion, long-term callus culture is an essential step used for *Agrobacterium*-mediated transformation (Eady et al., 2000; Kamata et al., 2011). To circumvent the unexpected growth retardation and vulnerability of the plants observed in this study, the development of new genome editing approaches that do not require a callus phase is highly desirable. Recently, in planta transformation, wherein the shoot apical meristem is targeted for CRISPR/Cas9 gene transfer, has successfully generated genome-edited plants (Imai et al., 2020; Kumagai et al., 2022; Sebiani-Calvo et al., 2024; Sasaki et al., 2025). For the production of tear-free onions using our approach, the application of in planta transformation technologies represents a promising future strategy.

## 5 Conclusions

In this study, we successfully generated *LFS* genome-edited onion plants, by *Agrobacterium*-mediated *CRISPR*/*Cas9* and gRNA transfer through the selection of elite callus lines with high transformation efficiency. For genome editing, we used the *Agrobacterium*-mediated gene delivery method, wherein calli induced from primary roots served as explants. As expected, LFS enzyme activity was reduced in the bulbs from genome-edited plants, thereby providing proof of concept for the potential generation of tear-free onions. However, poor growth of the regenerated plants and impaired bulb formation during soil cultivation, likely associated with long-term callus culture, remain critical challenges. Alternative genome editing methods for onion that do not require a callus phase may facilitate the production of tear-free onions with normal growth.

## Data Availability

The datasets presented in this study can be found in online repositories. The names of the repository/repositories and accession number(s) can be found in the article/**Supplementary Material**.
