# Supplementary material for: Generation of lachrymatory factor synthase-suppressed onion (Allium cepa L.) by Agrobacterium-mediated gene transfer for CRISPR/Cas9 genome editing
Source: Front Plant Sci. 2026 Sep 30;17:1949452. doi: 10.3389/fpls.2026.1949452 (PMC13662174; doi:10.3389/fpls.2026.1949452)
Supplement: Supplementary file 1 [file Table1.docx]

Supplementary Material

# 1 Supplementary Tables

Supplementary Table 1. Target sequences for *LFS* genome editing.

| Target | Sequence (5′ → 3′) PAM |
| --- | --- |
| #1 | GTAGTCGCTGATAGTGCTAACGG |
| #2 | AACGGAGCTCGAAAATGGAGCGG |
| #3 | CCAAATACAAAGCCAGAGCAAGC |
| #4 | CCTTCGTTGTCAGTCTGTGAACT |
| #5 | GGTTGTGTTCGCTACGTTAAAGG |
| #6 | GTGCTAACGGAGCTCGAAAATGG |
| #7 | ATACAAAGCCAGAGCAAGCATGG |

Protospacer adjacent motif (PAM) sequences are indicated in red.

Supplementary Table 2. Primers used in this study.

| Primer | Sequence (5′ → 3′) | Analyzed  target | Analysis | Expected  size |
| --- | --- | --- | --- | --- |
| LFS-F3 | CAATTCAGACTCACATTACGTTAT | #1, 2, 3 | NGS | 677 bp |
| LFS-R5 | ACACACAACACTCAGTCTTAC |  |  |  |
| sipLFS-F4 | CAAATACAAAGCCAGAGCAAGC | #4 | Nested PCR for  HMA | 164 bp |
| sipLFS-R4 | AAATTCCTCTTCTATTGGGTGCAT |  |  |  |
| sipLFS-F1 | GCCTTCGTTGTCAGTCTGTGAACT | #5 | Nested PCR for  CAPS　(*Mse*I) | 194 bp  (66, 128 bp) |
| sipLFS-R1 | CCGTGTAATCCTCGTACCCTGTAA |  |  |  |
| sipLFS-F2 | AAATCCTGGTGCACCTGC | #6 | Nested PCR for  CAPS (*Sac*I) | 106 bp  (46, 60 bp) |
| sipLFS-R2 | CCATGCTTGCTCTGGCTTTG |  |  |  |
| sipLFS-F3 | TAGTCGCTGATAGTGCTAACGG | #7 | Nested PCR for  CAPS (*Xcm*I) | 137 bp  (79, 58 bp) |
| sipLFS-R3 | TGACAACGAAGGCATGACC |  |  |  |

LFS, Lachrymatory factor synthase.

Supplementary Table 3. DeepSpCas9 scores of targets used in this study.

| Target | DeepSpCas9 score | Mutation detected |
| --- | --- | --- |
| #4 | 81.408 | ○ |
| #2 | 56.103 | ○ |
| #7 | 50.624 | ○ |
| #1 | 42.270 | ○ |
| #6 | 31.451 | × |
| #5 | 15.793 | ○ |
| #3 | 12.949 | × |

Detectable mutation outcome for each target is indicated as ○ (detected) or × (not detected).
